# School-level factors associated with the sustainment of weekly physical activity scheduled in Australian elementary schools: an observational study

**DOI:** 10.1186/s12889-022-13732-6

**Published:** 2022-07-23

**Authors:** Adam Shoesmith, Alix Hall, Luke Wolfenden, Rachel C. Shelton, Serene Yoong, Melanie Crane, Cassandra Lane, Nicole McCarthy, Christophe Lecathelinais, Nicole Nathan

**Affiliations:** 1 School of Medicine and Public Health, The University of Newcastle, 1 University Drive, Callaghan, NSW 2308 Australia; 2 Hunter New England Population Health, Hunter New England Local Health District, Locked Bag No. 10, Wallsend, NSW 2287 Australia; 3 Priority Research Centre for Health Behaviour, The University of Newcastle, University Drive, Callaghan, NSW Australia; 4 Hunter Medical Research Institute, New Lambton Heights, NSW Australia; 5 Department of Sociomedical Sciences, Mailman School of Public Health, Columbia University, New York, NY 10032 USA; 6 The Australian Prevention Partnership Centre, Sydney School of Public Health, The University of Sydney, Camperdown, NSW 2006 Australia

**Keywords:** Sustainability, Sustainment, Physical activity, Implementation, Scheduling, Schools, Interventions, Guidelines, Determinants, Factors

## Abstract

**Background:**

We aimed to: (1) identify school-level factors associated with the sustainment of weekly physical activity (PA) scheduled in elementary schools following withdrawal of effective implementation support; and (2) determine teacher’s perceived usefulness of suggested strategies for sustaining the scheduling of weekly PA.

**Methods:**

A secondary exploratory analysis was employed of data from the intervention arm (*n* = 31 schools) of a randomised controlled trial. Self-report survey data from 134 classroom teachers in New South Wales, Australia, collected following withdrawal of initial implementation support (follow-up T1) and six-months following completion of support (follow-up T2) were used. The outcomes of sustainment of weekly overall PA and energisers (short classroom PA breaks) scheduled were measured via teachers’ completion of a daily activity logbook, with results presented as the difference in mean minutes of PA and energisers scheduled at T1 and T2. An adapted version of the Program Sustainability Assessment Tool (PSAT) was used to measure capacity for program sustainability across seven key domains at follow-up T2. Linear mixed regressions were conducted to evaluate associations between school-level sociodemographic characteristics (e.g., school size, remoteness, and type), teacher-reported school factors (i.e., seven adapted PSAT domains) and the sustainment of PA and energisers scheduled across the school week. Perceived usefulness of 14 proposed sustainability strategies was measured via the teacher survey at follow-up T2 and reported descriptively.

**Results:**

No school-level factor was statistically associated with the sustainment of overall weekly PA or energisers scheduled. Teacher-reported factors in two PSAT domains – ‘strategic planning’ and ‘program evaluation’ were statistically negatively associated with the sustainment of weekly energisers scheduled (− 6.74, 95% CI: − 13.02; − 0.47, *p* = 0.036 and − 6.65, 95% CI: − 12.17; − 1.12, *p* = 0.019 respectively). The proposed support sustainability strategy – ‘provision of PA equipment packs that enable energisers or integrated lessons’ was perceived useful by the most teachers (85%).

**Conclusions:**

Further research is required to explore additional contextual-specific, and end-user appropriate factors associated with schools’ sustainment of weekly PA scheduled. This will help accurately inform the development of strategies to address these determinants and support the sustainment and long-term benefits of school-based health interventions more broadly.

**Supplementary Information:**

The online version contains supplementary material available at 10.1186/s12889-022-13732-6.

## Background

To improve children’s physical activity (PA) levels, the World Health Organization (WHO) has recommended the implementation of policies which specify the minimum amount of time schools are to deliver PA each week [1]. Systematic review evidence highlights the effectiveness of school-based policies that increase student moderate-to-vigorous PA, for example through physical education (PE) or other structured PA at school [2, 3]. Accordingly, governments internationally including several jurisdictions in Australia [4], Canada [5–7], Denmark [8], the United Kingdom [9] and the United States [10, 11] have developed school-based PA policies or guidelines stipulating the minimum number of minutes PA is to be provided across the school week. Despite the existence of such policies, many schools fail to schedule the required minutes of PA stipulated by these policies without external implementation support [5–14]. Numerous controlled trials have investigated strategies designed to facilitate schools’ delivery of PA interventions (e.g., centralise technical assistance and provide ongoing consultation, identify and prepare school champions, develop, and distribute educational materials, and change physical structure and equipment) [12, 13, 15]. These studies found significant improvements in the scheduling of weekly PE or PA, congruent with respective policy guidelines.

If the long-term public health benefits of school PA interventions, such as the PA policies described above, are to be realised, their sustainment is essential [16, 17]. Sustainment has been defined as ‘the continued use or delivery of an intervention in practice following cessation of external implementation support’ [18, 19]. However, evidence suggests the continued delivery of public health interventions post withdrawal of active support can be a challenge [20, 21]. A recent systematic review examining the sustainment of school-based public health interventions identified that none of the 18 included interventions were sustained in their entirety (i.e., all components) following the cessation of external implementation support (i.e., external start-up funding) [17]. Moreover, evidence suggests that when external implementation support for a health intervention is withdrawn, the quality of intervention delivery may reduce or cease to be delivered altogether, reducing its impact on desired health behaviours or outcomes [19, 20, 22].

To avoid attenuation of intervention effects and ensure ongoing, long-term delivery of PA by schools following withdrawal of active implementation support, strategies are needed to address key determinants of sustainment [21, 23, 24]. Understanding the specific determinants of intervention sustainment will enable researchers and practitioners to address barriers that impede long-term intervention delivery while also actively promoting factors that facilitate long-term delivery [17, 19–21]. Several systematic reviews highlight the possible determinants influencing the sustainment of health promoting interventions within the school setting [16, 17, 25]. For example, the most recent review found the most frequently identified factors influencing intervention sustainment from qualitative and quantitative data across 31 articles were: ‘the availability of facilities or equipment’, ‘continued executive or leadership support present’, and ‘team cohesion, support, or teamwork’ [25].

While these reviews provide important information on the possible determinants of intervention sustainment within schools more broadly, none of the included studies used a sustainability-specific theoretical framework to prospectively assess the multi-level (i.e., school-level or teacher-reported) factors associated with the sustainment of school-based health interventions. Consequently, the factors identified are not always consistent or easily synthesised, partially due to the wide variation in framework terminology, methods and measures used to classify determinants of sustainment in individual studies [16, 25]. Theoretically informed studies using specified and validated measures of sustainability determinants will enable accurate identification of determinants associated with sustainment of PA interventions scheduled within the school setting [19, 21]. This will also help inform the development, tailoring, refinement, and empirical testing of appropriate strategies to support their sustainment [19]. Identifying such strategies is however not merely informed by targeting influential determinants but understanding what sustainability strategies can be feasibly embedded within the school setting and succeed beyond the withdrawal of active implementation support. To ensure the longevity of such strategies and their effects on the longer-term maintenance and sustainment of weekly PA scheduled (i.e., > 6 months following cessation of implementation support), it is important to identify which strategies are perceived as useful by end-users (i.e., classroom teachers) [19, 26]. If strategies are not perceived as useful by end-users, they are less likely to be adhered to and less feasible to implement over time [26].

The current study contributes to improving our understanding of what is needed to support the sustainment of school-based interventions targeting PA scheduled by classroom teachers, once active support is withdrawn. Specifically, we aimed to: (1) identify school-level sociodemographic and teacher-reported school factors associated with the sustainment of weekly PA scheduling in elementary schools following withdrawal of effective implementation support, using a comprehensive theoretical and validated measure of sustainability determinants; and (2) determine teacher’s perceived usefulness of proposed sustainability strategies designed to support schools’ sustainment of weekly PA scheduled once delivery of the intervention had completed.

## Methods

### Context

This study reports on data from a cluster randomised controlled trial (RCT) which aimed to assess the effectiveness of a multi-strategy intervention – *‘Physically Active Children in Education (PACE)’*. PACE was designed to support classroom teachers’ scheduling of PA across the school week, in line with a mandatory state-level PA policy [13, 27]. This policy requires public schools in New South Wales (NSW) to incorporate 150 minutes per week of moderate, with some vigorous, PA for students in kindergarten to Grade 10 [28]. This may be achieved by delivery of PE, sport, or structured activity such as energisers (3–5 minute structured classroom PA breaks that require limited resource), and active lessons (PA integrated with other curricular subjects) [27–29]. To support schools’ delivery of the policy, PACE consisted of eight discrete implementation strategies that are described in detail elsewhere [12, 13, 27]. In summary, strategies included: centralise technical assistance and provide ongoing consultation, principal’s mandated change, identify and prepare school champions, develop implementation plans, conduct educational outreach visits, develop, and distribute educational materials, capture and share local knowledge, and change physical structure and equipment. At 12-month follow-up (immediately after withdrawal of initial implementation support), teachers at intervention schools scheduled significantly more weekly minutes of PA (an average of 44.2 minutes) (95% CI 32.8; 55.7, *p* < 0.001) than the control group [13]. The area of greatest improvement was observed in the scheduling of energisers, contributing to 52% (23.1 minutes) of the overall increase in weekly minutes of PA scheduled [13].

### Study design and setting

A secondary exploratory analysis was employed using data from two follow-up time points from the intervention arm (*n* = 134 classroom teachers from 31 elementary schools) of the PACE effectiveness RCT delivered in the Hunter New England (HNE) region of NSW Australia. HNE is a demographically and geographically diverse region, covering an area of approximately 130,000 km^2^ and including densely populated regions (i.e., metropolitan and regional hubs) as well as more geographically isolated areas (i.e., rural and remote locations) [30]. HNE is also socioeconomically diverse, with both areas of high wealth and areas of poverty [30]. The HNE region has a population of approximately 40,847 elementary school-aged children 5–12 years [31], with primary schools operating, on average, from 9 am to 3 pm Monday through Friday. Data for the current study were collected at follow-up time point one (T1) immediately following cessation of PACE implementation support (i.e., post intervention delivery at 12-months from baseline; October to December 2018), and follow-up time point two (T2) 6 months after PACE implementation support ended (i.e., 18-month following baseline; April to June 2019).

### Ethical considerations

The PACE trial was prospectively registered with the Australian New Zealand Clinical Trials Registry (ACTRN12617001265369). Ethical approval was obtained from the HNE Human Research Ethics Committee (no. 6/7/26/4.04), the Catholic Schools Office (no. 2012277), the University of Newcastle Human Research Ethics Committee (no. H-2008-0343), and NSW Department of Education (no. 2017184). This study conforms to the Strengthening the Reporting of Observational Studies in Epidemiology (STROBE) standards for reporting observational studies (see Additional file 1).

### Participants and recruitment

All government and Catholic elementary schools in the study region were considered eligible if they were not currently participating in another PA intervention or did not cater exclusively for children requiring specialist care [13, 27]. School principals were provided with a study information package and asked to provide written informed consent. Following principal consent, all classroom teachers were provided with a brief overview of the study purpose and invited to participate in a self-report survey [27]. Completion of the teacher survey was deemed consent. Classroom teachers who completed a paper survey at both time points were included in these analyses.

### Measures

#### Outcomes

##### Difference in mean weekly minutes of PA and energisers scheduled between completion of PACE implementation support and six-months following completion of PACE:

The primary outcome was the difference in mean minutes of PA scheduled by classroom teachers each day across the school week (i.e., 5 days) between follow-up T1 and follow-up T2. The outcome was measured via teacher’s completion of a daily activity logbook [27], which has established reliability and is frequently utilised in classroom-based obesity prevention interventions with high response rates (i.e., > 80%) [32–34]. The logbook included the time and occasions PA was allocated for PE, sport, energisers, or integrated lessons. Overall weekly PA was calculated by summing the time for each of these segments together. Data were included in the analyses if teachers provided complete data for the entire school week (i.e., 5 days). Reporting of the total number of minutes of PA for the week in excess of 250 minutes were capped, as values above this amount were considered highly unlikely given the context of Australian elementary schools and the Department of Education’s guidance of minimum time required for delivering other key learning areas [35]. As a secondary outcome we examined the difference in the mean minutes of energisers scheduled across the school week between follow-up T1 and follow-up T2, given the important contribution of energisers identified previously in increasing the overall scheduling of weekly PA [13].

##### Perceived usefulness of proposed strategies to support the sustainment of weekly PA scheduled following the withdrawal of implementation support:

Teacher’s perceived usefulness of 14 proposed discrete strategies designed to support the sustainment of weekly PA scheduled, following the withdrawal of PACE implementation support was assessed via the teacher survey at follow-up T2. Teachers were asked to indicate how useful they perceived each strategy on a five-point Likert scale, ranging from (1) ‘not useful at all’ to (5) ‘extremely useful’. These proposed strategies were developed by four implementation experts with school teaching experience, and four evaluation experts. Strategies were designed to address factors found to be influential to intervention sustainment [16–19, 25, 26]. An expert advisory group consisting of teachers, PA experts, implementation and evaluation specialists and government policy makers reviewed the list to ensure that strategies were relevant, practical, and feasible to be delivered by the local health district responsible for supporting schools’ delivery of the NSW PA policy. It is recognised that strategies required to support longer-term maintenance and sustainment (i.e., > 6 months following withdrawal of support) may differ from those strategies required during active implementation and immediate maintenance (i.e., < 6 months) [26]. Therefore the objective of obtaining this information was to inform the development of future sustainability strategies that may be required to support the ongoing, long-term sustainment of PA scheduled by schools following cessation of the active implementation support of PACE.

#### Independent variables

##### School-level sociodemographic determinants of sustainability:

Detail regarding school type (i.e., government or Catholic), school size, and postcode (used to generate school Socio-Economic Indexes for Areas [SEIFA], and remoteness) was obtained from the ‘my schools’ website [36]. These school-level sociodemographic factors have been identified or theorised to impact on intervention sustainment [37].

##### Classroom teacher demographics:

Classroom teacher demographic data were collected through the paper-based teacher surveys. Teachers were asked; age (in years), sex, grade level taught, if they were a specialist PE teacher, if they job share with another teacher (i.e., share one contract and split teaching of the same class with another teacher), their employment status (permanent, part-time, temporary, and casual), years teaching experience, and whether their school had a PA plan or policy.

##### Teacher-reported school determinants of sustainability:

Teacher-reported school factors theorised to impact on the sustainment of weekly PA scheduled were measured using the Program Sustainability Assessment Tool (PSAT). The PSAT is a measure of a public health program’s capacity for sustainability and was designed to evaluate important determinants that impact on the continued delivery of public health programs [38]. We recently adapted and psychometrically evaluated the PSAT for use in the elementary school setting (details published elsewhere) [39], which was then used in the current study. The adapted PSAT consists of 26-items across seven domains theorised to impact on program sustainment in the context of elementary schools, and include: strategic planning (3 items), environmental support (5 items), program adaptation (3 items), organisational capacity (5 items), program evaluation (3 items), funding stability (4 items) and communications (3 items) [39] (see Additional file 2). The domains used in the current study reflect the same domains identified in the confirmatory factor analysis conducted in the validation paper [39]. These factors were measured from the perspective of classroom teachers via the survey at follow-up T2.

The adapted PSAT showed strong evidence of internal reliability, with Cronbach’s alpha values ranging from 0.77 to 0.92 [39]. However, evidence for structural validity was mixed and was lacking for convergent validity [39]. The adapted PSAT was included in the follow-up T2 survey. Using a seven-point Likert scale ranging from ‘strongly disagree’ (1) to ‘strongly agree’ (7), teachers were asked to indicate their level of agreement with each item relating to program sustainability. Domain scores were calculated by summing the items in each of the PSAT domains and dividing by the number of non-missing items. Domain scores were only calculated for teachers who answered a minimum of 50% of items from each domain. Domains with lower average scores indicated areas where capacity for sustainability could be improved [38].

### Statistical analysis

All analyses were performed in SAS version 9.3 (SAS Institute, Cary, North Carolina, USA). Descriptive statistics, including means, standard deviations (SD), medians and quartiles (Q1 and Q3) for continuous data, and frequencies and percentages for categorical data, were used to describe school and classroom teacher characteristics (see Tables 1 and 2). School postcode was used to categorize school locality as either ‘rural’ (outer regional, remote, and very remote areas) or ‘urban’ (regional cities and inner regional areas) based upon the Australian Bureau of Statistics Australian Statistical Geography Standard [40]. Schools with postcodes in the top 50% of NSW postcodes, based on the disadvantage index of the Australian Bureau of Statistics SEIFA, were categorized as ‘higher socio-economic areas’, while those in the lower 50% were categorized as ‘lower socio-economic areas’ [40].

**Table 1:** Demographic characteristics of participating schools

Demographic characteristic	Total (n)
**Total schools**	***n*** **= 31**
**School size (mean no. of students), n (sd)**	282.10 (145.07)
**Range of classrooms per school (median), (minimum; maximum)**	10 (3; 22)
**School type, n (%)**	
Government	21 (67.74%)
Catholic	10 (32.26%)
**Socio-economic Indexes for Areas (SEIFA), n (%)**	
Most disadvantaged	20 (64.52%)
Least disadvantaged	11 (35.48%)
**Remoteness, n (%)**	
Inner / outer regional Australia	13 (41.94%)
Major cities of Australia	18 (58.06%)

**Table 2:** Demographic characteristics of teachers completing both time points and those excluded from the analyses

Demographic characteristic	Total included (n)	Total excluded (n)	***p***-value
**Total classroom teachers**	***n*** **= 99**	***n*** **= 35**	
**Age (mean), yrs (sd)**^a^	40.82 (10.38)	41.00 (11.51)	0.64
**Sex, n (%)**^a^			
Male	17 (17%)	3 (9%)	0.38
Female	81 (83%)	32 (91%)	
**Teaching role, n (%)**^a^			
Yes	98 (100%)	30 (97%)	0.92
No	0 (0%)	1 (3%)	
**Specialist PE teacher, n (%)**^a^			
Yes	4 (4%)	0 (0%)	0.97
No	92 (96%)	32 (100%)	
**Job share, n (%)**^a^			
Yes	18 (19%)	8 (26%)	0.19
No	79 (81%)	23 (74%)	
**Employment status, n (%)**^a^			
Full time	85 (88%)	30 (94%)	0.49
Part time/Casual	12 (12%)	2 (6%)	
**Number of years teaching (mean), n (sd)**^a^	15.06 (10.32)	15.00 (9.83)	0.76
**School PA policy, n (%)**^a^			
Yes	68 (84%)	23 (85%)	0.38
No	13 (16%)	4 (15%)	

^a^Totals may not equal total sample size due to missing values

#### Aim 1: factors associated with the sustainment of weekly PA scheduling

The difference in mean weekly minutes of PA and energisers scheduled between follow-up T1 and T2 were calculated and represented the dependent variable (see Table 3). An increase in the scheduled minutes of PA and energisers was represented by a positive difference between time points, indicating sustained delivery of PA or energisers. Linear mixed regression analyses examined the associations between school-level sociodemographic factors (i.e., school size, SEIFA, remoteness, and type) and classroom teacher-reported factors (i.e., having a school PA plan or policy, and each PSAT domain scores) with the sustainment of overall PA (primary outcome) and energisers (secondary outcome) scheduled across the school week. The linear mixed regression models included a random intercept for school to account for the clustered sample and were adjusted for potential confounders (years of teaching, whether teacher was PE trained and employed full time) by including them as fixed effects. The unadjusted and adjusted regression coefficients and their corresponding 95% confidence intervals (CI) are presented in Table 4, including the *p*-value from the adjusted model. An alpha level of 5% was used to determine a significant association between factors and the level of sustainment of overall weekly PA and energisers scheduled.

**Table 3:** Outcome measures of weekly PA and energisers scheduled between follow-up T1 and T2 and PSAT domain scores at T2

Outcome	Total (n)	Mean (sd)	Median (Q1; Q3)	ICC
**Difference in scheduling of PA between follow-up time points (T1 and T2) – mean minutes per school week**				
Overall PA	99	1.65 (59.63)	-5 (− 30; 25)	0.125
Energisers	99	7.64 (31.82)	10 (−10; 25)	0.254
**PSAT domain scores (measured at T2)**^**a**^ **– mean score**				
Strategic planning	98	5.04 (1.06)	5.00 (4.67; 6.00)	0.311
Environmental support	98	5.18 (0.85)	5.20 (4.80; 5.80)	0.293
Program adaptation	99	5.14 (0.92)	5.33 (4.67; 6.00)	0.121
Organisational capacity	99	5.33 (0.92)	5.29 (4.75; 5.75)	0.253
Program evaluation	99	4.36 (1.16)	4.25 (3.75; 5.00)	0.271
Funding stability	98	4.83 (0.99)	4.75 (4.25; 5.50)	0.320
Communications	99	4.74 (0.92)	4.67 (4.00; 5.33)	0.291

^a^The possible range of PSAT domain scores is 1 to 7

**Table 4:** Associations between factors and difference in weekly minutes of PA and energisers implemented between time points

Factor	Total weekly minutes of PA implemented				Total weekly minutes of energisers implemented		
	Total (n)	Unadjusted coefficient (95%CI)^a^	Adjusted coefficient (95%CI)^a^	*p*-value^b^	Unadjusted coefficient (95%CI)^a^	Adjusted coefficient (95%CI)^a^	*p*-value^b^
***School-level sociodemographic factors***							
School size	99	−0.02 [− 0.13; 0.09]	− 0.03 [− 0.14; 0.09]	0.60	−0.03 [− 0.10; 0.03]	−0.02 [− 0.10; 0.05]	0.48
School SEIFA	99						
Most disadvantaged		−12.82 [−49.21; 23.58]	−11.39 [− 49.15; 26.37]	0.52	−7.74 [−29.72; 14.24]	−10.92 [−34.07; 12.24]	0.33
Least disadvantaged (R1)^		–	–		–	–	
School remoteness	99						
Major cities		−3.81 [−43.24; 35.61]	−6.15 [− 46.89; 34.58]	0.73	−8.98 [− 32.27; 14.31]	− 6.81 [− 31.88; 18.25]	0.56
Inner / outer regional / remote Australia (R1)^		–	–		–	–	
School type	99						
Catholic		16.64 [−21.11; 54.40]	18.91 [−20.01; 57.82]	0.31	19.33 [−0.74; 39.39]	19.77 [−1.70; 41.24]	0.07
Government (R1)^		–	–		–	–	
***Teacher-reported school-level factors***							
School PA plan or policy	89						
Yes		−6.46 [−47.36; 34.44]	−13.98 [−57.51; 29.55]	0.52	− 5.32 [−25.92; 15.28]	− 6.90 [− 28.62; 14.81]	0.53
No (R1)^		–	–		–	–	
Strategic planning	95	−7.29 [− 19.35; 4.78]	−7.39 [− 20.17; 5.39]	0.25	−6.53 [− 12.69; −0.36]	−6.74 [− 13.02; − 0.47]	**0.036**
Environmental support	96	−8.18 [− 23.24; 6.88]	−8.62 [− 24.83; 7.60]	0.29	−3.01 [− 10.72; 4.71]	− 3.31 [− 11.47; 4.84]	0.42
Program adaptation	96	1.21 [− 12.32; 14.74]	1.64 [− 13.02; 16.30]	0.82	−2.21 [− 9.08; 4.65]	−1.53 [− 8.65; 5.58]	0.67
Organisational capacity	96	0.38 [− 13.97; 14.74]	0.38 [−15.52; 16.28]	0.96	−3.04 [− 10.37; 4.29]	−2.72 [− 10.52; 5.08]	0.49
Communications	96	−4.38 [− 17.99; 9.22]	−5.72 [− 20.54; 9.09]	0.44	− 5.55 [− 12.53; 1.42]	−5.16 [− 12.55; 2.24]	0.17
Program evaluation	96	−9.49 [− 20.02; 1.03]	−9.24 [− 20.41; 1.93]	0.10	−6.93 [− 12.29; − 1.57]	−6.65 [− 12.17; −1.12]	**0.019**
Funding stability	96	1.07 [− 11.97; 14.12]	1.66 [− 12.00; 15.32]	0.81	−4.50 [− 11.08; 2.08]	−3.95 [− 10.67; 2.78]	0.25

^a^Linear mixed regression analyses were used to calculate coefficient and *p*-values. Coefficients correspond to the influence of a unit increase in each factor on the difference in PA and energisers implemented across the school week between 12 and 18-month follow-up. Results are reported as adjusted and unadjusted regression coefficients with corresponding 95% confidence intervals. Adjusted analyses included a random intercept for school and were adjusted for number of years teaching, whether they are a PE teacher and whether they are employed full time

^R1 indicates the reference group for dichotomous variables

^b^*p*-value listed is for the coefficient from the adjusted model. Significance was set at *p* ≤ 0.05 with bolded *p*-values indicating significance

#### Aim 2: perceived usefulness of proposed sustainability strategies

Descriptive statistics were used to describe the proportion of teachers that indicated whether each strategy was perceived as ‘useful’ for sustaining the scheduling of weekly PA (collapsed survey responses of ‘useful’ and ‘extremely useful’). Strategies were ranked from highest to lowest frequency, in order to determine the most useful sustainability strategies perceived by teachers (see Table 5).

**Table 5:** Teachers that perceived proposed sustainability strategies as useful

Sustainability strategy proposed	Number of respondents (n)	Total teachers who agreed strategy was useful, n (%)
PA equipment packs that enable energisers or integrated lessons	*n* = 92	78 (85%)
In the event of new staff at the school, an information pack to upskill new staff about the policy and how to implement it within their classroom	*n* = 92	72 (78%)
A whole school PA plan outlining the school’s steps to ensure the PA policy is met in the future	*n* = 91	71 (78%)
Professional learning modules to assist staff in meeting the PA policy delivered face-to-face	*n* = 92	70 (76%)
In the event of staff turnover, a formal hand-over document & information pack to support new school champions take on the role of supporting the PA policy implementation in the school	*n* = 92	67 (73%)
An annual whole staff or stage meeting to review implementation of the policy and share ideas to help ensure implementation of the policy is sustained	*n* = 92	65 (71%)
Professional learning modules to assist staff in meeting the PA policy delivered online	*n* = 92	64 (70%)
Biannual webinars on ideas of how to integrate PA into your class schedule	*n* = 90	62 (69%)
A face to face program booster session for school champions every two years	*n* = 92	62 (67%)
Our school executive monitoring that the PA policy is being met when reviewing our class timetables	*n* = 92	61 (66%)
Scheduling 150 minutes (DoE schools) / 120 minutes (CSO schools) of PA being included in the school’s strategic plan and reported on in annual reports	*n* = 92	56 (61%)
Unlimited telephone or email support from a program support officer	*n* = 92	52 (57%)
Our school director prompting our executive about meeting the PA policy during an annual meeting with school	*n* = 91	34 (37%)
Including a standing agenda item for all staff meetings to discuss ideas of how to best implement and monitor our school’s adherence to the PA policy	*n* = 92	34 (37%)

Strategies are listed in descending order from those perceived as most useful least useful

## Results

### Participation and sample characteristics

School and teacher demographic data are provided in Tables 1 and 2. Of the 31 participating schools, 134 classroom teachers completed surveys at both follow-up time points (T1 and T2). Of these, 110 teachers (82%) provided valid scheduling data across both time points (i.e., 5 days), and 99 teachers (74%) provided both valid scheduling and PSAT data, and thus were included in the analyses (see Table 2). There were no significant differences in the demographic characteristics of teachers with valid scheduling and PSAT data (*n* = 99), and those excluded from the analyses (*n* = 35) (see Table 2). Of teachers with valid data, 83% were female with a mean age of 40.82 (SD = 10.38) years.

On average, from follow-up T1 to T2 teachers increased their overall weekly scheduling of PA by 1.65 minutes (95% CI: − 10.24; 13.55, *p* = 0.78) and energisers by 7.64 minutes (95% CI: 1.29; 13.98, *p* = 0.02) (see Table 3). Overall, teachers reported PSAT domain scores at T2 ranging from 4.36 (SD = 1.16) (program evaluation domain) to 5.33 (SD = 0.92) (organisational capacity domain).

### Associations between school-level sociodemographic and teacher-reported school factors and the sustainment of weekly PA scheduling

None of the school-level sociodemographic or teacher-reported school factors assessed were found to be statistically significantly associated with a difference in teacher’s mean minutes of PA scheduled across the school week between follow-up T1 and T2 (see Table 4). Of the PSAT scores, four domains possessed a negative association with the sustainment of weekly PA scheduled and three possessed a positive association, although no adapted PSAT domains were statistically significantly associated.

For the secondary outcome of difference in mean minutes of energisers scheduled between follow-up T1 and T2, all adapted PSAT domain scores possessed a negative association. In the adjusted regression model, two of the adapted PSAT domain scores – ‘strategic planning’ and ‘program evaluation’ – were found to be statistically negatively associated with the sustainment of weekly energisers scheduled (see Table 4). For every unit increase in the domain score for ‘strategic planning’, the total minutes of energisers scheduled across the school week from T1 to T2 decreased by approximately 6.74 minutes (95% CI: − 13.02; − 0.47, *p* = 0.036). Similarly, for every unit increase in the domain score for ‘program evaluation’, the difference in the total minutes of energisers scheduled across the school week from T1 to T2 decreased by approximately 6.65 minutes (95% CI: − 12.17; − 1.12, *p* = 0.019) (see Table 4). We conducted a sensitivity analysis to assess the robustness of the results for teachers who completed all three time points (i.e., baseline, follow-up T1 and T2). No significant changes were observed with regards to factors identified as being associated with a difference in scheduling of overall PA and energisers from follow-up T1 to T2 in this sample of teachers (see Additional file 3).

### Perceived usefulness of proposed sustainability strategies

Table 5 details the frequency and percentage of teachers that perceived the proposed sustainability strategies, as useful in descending order. The most useful strategies perceived by teachers to support their delivery long-term, related to the resources, skills/knowledge, and training available. Specifically, ‘provision of PA equipment packs that enable energisers or integrated lessons’, was reported as useful by most teachers (*n* = 78 [85%]). This was followed by the ‘provision of an information pack to upskill new staff about the policy and how to implement it within their classroom’, and ‘creating a whole school PA plan outlining the school’s steps to ensure the PA policy is met in the future’, as reported by 72 (78%) and 71 (78%) teachers, respectively. The strategies perceived as ‘useful’ by the least number of teachers were: ‘including a standing agenda item for all staff meetings to discuss ideas of how to best implement and monitor our school’s adherence to the PA policy’ (*n* = 34 [37%]); and ‘school director prompting our executive about meeting the PA policy during an annual meeting with school’ (*n* = 34 [37%]); followed by ‘provision of unlimited telephone or email support from a program support officer’ (*n* = 52 [57%]).

## Discussion

The current study contributes to improving our understanding of what is needed to support the sustainment of health interventions in schools. Specifically, we analysed school-level sociodemographic and teacher-reported school factors associated with the sustainment of weekly PA and energiser scheduling after the withdrawal of effective implementation support [13, 27], using a comprehensive theoretically informed measure of sustainability determinants. The study found that although teacher’s scheduling of weekly PA and energisers was on average sustained over this six-month period – no school-level sociodemographic characteristic was statistically associated with this sustainment. Teacher-reported school factors in two PSAT domains (strategic planning and program evaluation) were statistically negatively associated with the sustainment of weekly energisers scheduled, which was the opposite direction to what we hypothesised. We also determined teacher’s perceived usefulness of proposed strategies to assist schools in sustaining their scheduling of weekly PA once delivery of the PACE intervention had completed. The most useful strategies perceived by classroom teachers to assist with the sustainment of weekly PA scheduling, related to the provision of resources, skills/knowledge, and training available.

Findings suggest that the sustainment of PA scheduled in schools may be independent of sociodemographic school-level characteristics, given that no school-level characteristic hypothesised, such as school type, size, SEIFA, or remoteness were statistically associated with teacher’s sustainment of PA. This finding is consistent with other international studies [37, 41, 42]. For example, a 2018 prospective longitudinal study observed that school characteristics were not predictive of sustained delivery of *‘School-wide Positive Behavioural Interventions and Supports’* in the U. S [37]. Further, a 2019 cross-sectional study examining how school context, principal characteristics, and program attributes were associated with the institutionalisation of *‘Bluearth Foundation’s Active Schools’* program in Australian elementary schools, found that no school demographics were associated with program institutionalisation [42]. These findings suggest that intervention sustainment may be more influenced by other higher-level outer contextual factors (e.g., socio-political context, external funding, and leadership) or inner contextual factors (e.g., school climate/culture, capacity, and executive support) more so than demographics [19]. Given school-level sociodemographic characteristics (i.e., type, SEIFA or remoteness) are more difficult to change, this may indicate that the organisational factors that may be important sustainability determinants are also more conducive to change.

The current study also found that no teacher-reported school factors (PSAT domain scores) were statistically associated with the sustainment of weekly PA scheduled, and five of the seven domain scores were not statistically associated with the sustainment of energisers scheduled across the school week. These inconclusive findings are surprising given the use of a validated, sustainability-specific measure [39]. Statistically negative associations were identified between teacher-reported PSAT strategic planning factors (i.e., using processes that guide program direction, goals, and strategies) and program evaluation factors (i.e., assessing program data to inform planning and document results) and the sustainment of weekly energisers scheduled. This is contrary to previous study findings which have also examined these associations at the staff or practice (intervention) level [43, 44]. For example, McIntosh et al. identified that the strongest predictor of schools’ sustained delivery of *‘School-wide Positive Behavioural Interventions and Supports’* at 3 years was “better team use of data for decision making” in Year 1 [37, 45]. Similarly, a 2012 quantitative study by Coffey and Horner who surveyed conditions leading to the sustainability of *‘School-wide Positive Behavioural Interventions and Supports’* described above, found that the strongest predictor for sustainment was ‘use of data for decision making’ [41]. Our findings however conversely indicate that the increased use of data for evaluation was statistically associated with a decrease in the weekly scheduling of energisers between time points.

It is possible these discrepancies between current study findings and previous studies are impacted by the psychometric tool used to measure determinants of intervention sustainment across studies. In the current study we used the adapted the PSAT which is validated for use within the elementary school setting [39]. However, during the adaptation of the PSAT, authors observed mixed evidence of validity, particularly convergent validity, where there was no evidence of an association between the PSAT domains and scheduling of weekly PA at 18-month follow-up from baseline [39]. This may indicate that the adapted PSAT does not comprehensively cover the sustainability determinants of school-based interventions. One of the potential contributing factors to this measure lacking evidence of validity, is that teachers may not possess authority over, or have adequate knowledge of, the higher-level organisational structure/process and external factors that form a large focus of the PSAT items (e.g., capacity building, funding, or external support) [38, 39]. Instead, it may be more appropriate for frontline teachers to complete items covering factors they may have more accurate knowledge of (e.g., self-efficacy, motivation, skill/level of training, and feasibility/appropriateness of intervention delivery); and executives report on higher-level organisational factors (e.g., policy landscape, funding stability, and external partnership support). Further research is recommended to develop and empirically test valid, reliable, psychometrically robust, pragmatic, and specified measures of sustainability determinants tailored to multiple end-users (i.e., answered by executives and frontline staff separately) that are appropriate across a broad range of interventions [46]. This may enable a more comprehensive understanding of what specific determinants need to be addressed to support the sustainment of school-based health interventions [19, 46, 47].

This study also sought to explore strategies that may be useful in supporting teachers’ sustainment of weekly PA scheduling following the withdrawal of implementation support. Our findings indicate that the strategies perceived by the majority of teachers as useful in this regard were related to the resources, skills/knowledge and training available to support their delivery long-term. These align with and are likely to address some of the most prevalent barriers to the sustainment of school-based health behaviour interventions, such as poor availability of facilities, resources, equipment, and training opportunities [16, 17, 25]; and therefore, should be considered when planning sustainability support for such interventions. Comparatively, fewer teachers perceived training sessions, monitoring and feedback, and ongoing contact or support from external program providers as useful strategies. It appears within the current sample, whilst teachers are willing to acquire the skills and resources to enable the sustainment of weekly PA scheduling, they do not perceive themselves to be reliant on the ongoing external and intensive support from the program team. This suggests that teachers may find it appropriate to be offered less intensive strategies to ensure continued intervention delivery. In the wider evidence-base however, it is acknowledged that research evaluating sustainment strategies has been limited [26]. Few studies have empirically examined the use, effectiveness, and acceptability of strategies to sustain the delivery of interventions within the school setting [21, 26]. In addition, given sustainability has only ever been assessed at most up to 2 years post-implementation support [20], little is known regarding the longevity of strategies and what may be relevant beyond this time period, in supporting the sustainment of such health interventions. Further empirical work is needed, in consultation with end-users responsible for intervention delivery, to determine the most effective, feasible, acceptable, end-user tailored strategies that are intervention-specific, to support the ongoing sustainment of evidence-based health interventions in schools.

### Limitations

The findings of this study should be acknowledged in the context of its limitations. First, as the PSAT scores were collected at one time-point only, and these determinants were not assessed prior to the main outcome (change in minutes of weekly PA scheduled), causation and temporality cannot be inferred. Additional prospective, longitudinal, and experimental studies are required to assess the causal association between school-level and teacher-reported factors and the sustainment of weekly PA scheduling in the school setting. Second, the sustainment of weekly PA scheduling was measured across two time periods conducted over a short six-month timeframe. This provides some indication of sustainment, however, to comprehensively assess long-term sustainment ideally requires assessment over years [19]. Future longitudinal studies should be conducted, with data collected at multiple time points at longer follow-up intervals post cessation of active implementation support (e.g., 12, 18, 24-months). This would enable a more comprehensive understanding of any longer-term sustainment or possible attenuation in PA scheduled, in addition to the factors that may contribute to this. Third, our findings may be influenced by the previous delivery of the initial implementation strategy. The adapted PSAT examines schools’ capacity to sustain, and many of these factors were targeted in our initial implementation trial (e.g., school champions, executive support, and provision of resources). Given T2 follow-up in the current study was conducted 6 months following cessation of implementation support, these factors may still be active in schools. However, it is expected that some of these factors may change following a longer time period, thus helping to identify which schools are likely to have capacity to sustain long-term. Fourth, included data were restricted to teachers who completed surveys at both time points, which may contribute to any selection bias in this sample – whereby those not scheduling weekly PA at follow-up T1 may be more likely to be lost to follow-up T2. Fifth, the difference in mean weekly minutes of PA and energisers scheduled between T1 and T2 relied on self-report data via teachers’ daily logbook. This method was selected based on use in previous obesity-prevention trials [32–34], and analogous evidence suggesting such measures may represent a reliable and pragmatic measure of PA delivery in this the school setting [32–34]. However, such measures are at risk of social desirability and recall bias which may lead to over reporting in teacher’s scheduling of PA. Lastly, the multiple testing of a large number of characteristics in the regression models, may have resulted in false positive (i.e., type I error) findings.

## Conclusions

These findings contribute to improving broad understanding of what multi-level factors may need to be addressed; and which sustainability strategies may support the sustainment of school-based interventions targeting healthy behaviours, specifically in relation to weekly PA scheduling (i.e., provision of resources, skills/knowledge, and training available). Additional research is required to explore contextually specific and end-user appropriate factors associated with schools’ sustainment of weekly PA scheduling, using psychometrically-robust, valid, and reliable measures. This will ensure a more comprehensive understanding of what determinants need to be addressed and help accurately inform the development of strategies to support the sustainment and continued benefit of school-based health interventions long-term.

## Supplementary Information

**Additional file 1.** STROBE Statement – checklist of items that should be included in reports of observational studies.

**Additional file 2.** Adapted PSAT domains, definitions and items.

**Additional file 3.** Associations between factors and difference in weekly minutes of PA and energisers implemented between follow-up T1 and T2 for teachers who completed all time points.

## Data Availability

This study does not have approval to publicly share individual level data. However, the data and study materials that support the findings of this study are available from the corresponding author upon reasonable request and subject to appropriate and relevant approvals being granted.

## Abbreviations

WHO: World Health Organisation
PE: Physical education
RCT: Randomised controlled trial
PACE: Physically Active Children in Education
HNE: Hunter New England
NSW: New South Wales
PSAT: Program Sustainability Assessment Tool
SEIFA: Socio-Economic Indexes for Areas
SD: Standard deviation
IQR: Interquartile range

## References

[1] World Health Organization (WHO) (2018). Global action plan on physical activity 2018–2030: more active people for a healthier world.

[2] Pate RR, Trilk JL, Byun W, Wang J (2011). Policies to increase physical activity in children and youth. J Exerc Sci Fit.

[3] Brennan LK, Brownson RC, Orleans CT (2014). Childhood obesity policy research and practice: evidence for policy and environmental strategies. Am J Prev Med.

[4] Stylianou M, Walker JL (2018). An assessment of Australian school physical activity and nutrition policies. Aust N Z J Public Health.

[5] Naylor PJ, McKay HA (2009). Prevention in the first place: schools a setting for action on physical inactivity. Br J Sports Med.

[6] Nettlefold L, McKay H, Warburton D (2011). The challenge of low physical activity during the school day: at recess, lunch and in physical education. Br J Sports Med.

[7] Mâsse LC, Naiman D, Naylor PJ (2013). From policy to practice: implementation of physical activity and food policies in schools. Int J Behav Nutr Phys Act.

[8] Johansen DLN, Christensen BFN, Fester M (2018). Results from Denmark’s 2018 report card on physical activity for children and youth. J Phys Act Health.

[9] Harrington DM, Belton S, Coppinger T (2014). Results from Ireland’s 2014 report card on physical activity in children and youth. J Phys Act Health.

[10] Thompson HR, Linchey J, Madsen KA (2013). Peer reviewed: are physical education policies working? A snapshot from San Francisco, 2011. Prev Chronic Dis.

[11] Carlson JA, Sallis JF, Chriqui JF (2013). State policies about physical activity minutes in physical education or during school. J Sch Health.

[12] Nathan NK, Sutherland RL, Hope K (2020). Implementation of a school physical activity policy improves student physical activity levels: outcomes of a cluster-randomized controlled trial. J Phys Act Health.

[13] Nathan N, Hall A, McCarthy N, et al. Multi-strategy intervention increases school implementation and maintenance of a mandatory physical activity policy: outcomes of a cluster randomised controlled trial. Br J Sports Med Published Online First: 26 May. 2021. 10.1136/bjsports-2020-103764. 10.1136/bjsports-2020-103764 PMC8938653 34039583

[14] Webster CA, Caputi P, Perreault M (2013). Elementary classroom teachers’ adoption of physical activity promotion in the context of a statewide policy: an innovation diffusion and socio-ecologic perspective. J Teach Phys Educ.

[15] Sallis JF, McKenzie TL, Alcaraz JE, et al. The effects of a 2-year physical education program (SPARK) on physical activity and fitness in elementary school students. Sports, play and active recreation for kids. Am J Public Health. 1997;87(1328–34). 10.2105/AJPH.87.8.1328. 10.2105/ajph.87.8.1328 PMC1381094 9279269

[16] Cassar S, Salmon J, Timperio A (2019). Adoption, implementation and sustainability of school-based physical activity and sedentary behaviour interventions in real-world settings: a systematic review. Int J Behav Nutr Phys Act.

[17] Herlitz L, MacIntyre H, Osborn T, Bonell C (2020). The sustainability of public health interventions in schools: a systematic review. Implement Sci.

[18] Moore JE, Mascarenhas A, Bain J, Straus SE (2017). Developing a comprehensive definition of sustainability. Implement Sci.

[19] Shelton RC, Cooper BR, Stirman SW (2018). The sustainability of evidence-based interventions and practices in public health and health care. Annu Rev Public Health.

[20] Wiltsey Stirman S, Kimberly J (2012). The sustainability of new programs and innovations: a review of the empirical literature and recommendations for future research. Implement Sci.

[21] Bodkin A, Hakimi S (2020). Sustainable by design: a systematic review of factors for health promotion program sustainability. BMC Public Health.

[22] Shediac-Rizkallah MC, Bone LR (1998). Planning for the sustainability of community-based health programs: conceptual frameworks and future directions for research, practice and policy. Health Educ Res.

[23] Chambers DA, Glasgow RE, Stange KC (2013). The dynamic sustainability framework: addressing the paradox of sustainment amid ongoing change. Implement Sci.

[24] Koorts H, Eakin E, Estabrooks P (2018). Implementation and scale up of population physical activity interventions for clinical and community settings: the PRACTIS guide. Int J Behav Nutr Phys Act.

[25] Shoesmith A, Hall A, Wolfenden L (2021). Barriers and facilitators influencing the sustainment of health behaviour interventions in schools and childcare services: a systematic review. Implement Sci.

[26] Hailemariam M, Bustos T, Montgomery B (2019). Evidence-based intervention sustainability strategies: a systematic review. Implement Sci.

[27] Nathan N, Wiggers J, Bauman AE (2019). A cluster randomised controlled trial of an intervention to increase the implementation of school physical activity policies and guidelines: study protocol for the physically active children in education (PACE) study. BMC Public Health.

[28] NSW Government (2015). Rationale for change; sport and physical activity policy- revised 2015. NSW Department of Education and Communities.

[29] Webster CA, Russ L, Vazou S (2015). Integrating movement in academic classrooms: understanding, applying and advancing the knowledge base. Obes Rev.

[30] Wiggers J, Wolfenden L, Campbell E (2013). Good for kids, good for life 2006–2010 evaluation report.

[31] Australian Bureau of Statistics (2016). 2016 Census Quick Stats.

[32] Bessems KM, Van Assema P, Martens MK (2011). Appreciation and implementation of the Krachtvoer healthy diet promotion programme for 12- to 14- year-old students of prevocational schools. BMC Public Health.

[33] van Nassau F, Singh AS, van Mechelen W (2013). Exploring facilitating factors and barriers to the nationwide dissemination of a Dutch school-based obesity prevention program “DOiT”: a study protocol. BMC Public Health.

[34] Cradock AL, Barrett JL, Carter J (2014). Impact of the Boston active school day policy to promote physical activity among children. Am J Health Promot.

[35] NSW Government (2020). K–6 curriculum requirements.

[36] Australian Curriculum Assessment and Reporting Authority (ACARA) (2020). My school website.

[37] McIntosh K, Mercer SH, Nese RNT (2018). Factors predicting sustained implementation of a universal behavior support framework. Educ Res.

[38] Luke DA, Calhoun A, Robichaux CB (2014). The program sustainability assessment tool: a new instrument for public health programs. Prev Chronic Dis.

[39] Hall A, Shoesmith A, Shelton RC (2021). Adaptation and validation of the program sustainability assessment tool (PSAT) for use in the elementary school setting. Int J Environ Res Public Health.

[40] Australian Bureau of Statistics (2016). Technical Paper: Socio-Economic Indexes for Areas.

[41] Coffey JH, Horner RH (2012). The sustainability of schoolwide positive behavior interventions and supports. Except Child.

[42] Bourke M, Hilland TA, Craike M (2020). Factors associated with the institutionalization of a physical activity program in Australian elementary schools. Transl Behav Med.

[43] Abi Nader P, Hilberg E, Schuna JM Jr, et al. Association of teacher-level factors with implementation of classroom-based physical activity breaks. J Sch Health. 2019. 10.1111/josh.12754. 10.1111/josh.12754 30937920

[44] Webster C, Erwin H, Parks M (2013). Relationships between and changes in preservice classroom teachers’ efficacy beliefs, willingness to integrate movement, and perceived barriers to movement integration. Phys Educ.

[45] McIntosh K, MacKay LD, Hume AE (2011). Development and initial validation of a measure to assess factors related to sustainability of school-wide positive behavior support. J Posit Behav Interv.

[46] Moullin JC, Sklar M, Green A (2020). Advancing the pragmatic measurement of sustainment: a narrative review of measures. Implement Sci Commun.

[47] Mettert K, Lewis C, Dorsey C, et al. Measuring implementation outcomes: an updated systematic review of measures’ psychometric properties. Implement Res Prac. 2020. 10.1177/2633489520936644. 10.1177/2633489520936644 PMC9924262 37089128

