# Supplementary material for: School-level factors associated with the sustainment of weekly physical activity scheduled in Australian elementary schools: an observational study
Source: BMC Public Health. 2022 Jul 23;22:1408. doi: 10.1186/s12889-022-13732-6 (PMC9308175; doi:10.1186/s12889-022-13732-6)
Supplement: Supplementary file 2 — Additional file 2. Adapted PSAT domains, definitions and items. [file 12889_2022_13732_MOESM2_ESM.docx]

**Additional file 2.** Adapted PSAT domains, definitions and items

| **PSAT domain** | **Domain definition** | **Items** |
| --- | --- | --- |
| Strategic planning | Using processes that guide your program’s direction, goals, and strategies | My school has a sustainability plan (e.g., to continue the scheduling of the recommended minutes of physical activity long-term). |
|  |  | My school’s goals to maintain the scheduling of physical activity are understood by all stakeholders (e.g., teachers, school champions, principals). |
|  |  | My school clearly outlines roles and responsibilities to schedule physical activity for all stakeholders (e.g., teachers, school champions, principals). |
| Environmental support | Having a supportive internal and external climate for your program | There are champions within the school advocating for the scheduling of physical activity *(a champion is someone who supports and advocates the policy, this may be your school executive or a teacher within the school).* |
|  |  | There are champions within the school with the ability to get resources for the scheduling of physical activity. |
|  |  | My school has support from within the broader organisation i.e. DoE/ CSO for the scheduling of physical activity. |
|  |  | My school has support from outside our education department/office to help the scheduling of physical activity. |
|  |  | The scheduling of physical activity for students at my school has strong public and community support. |
| Program adaptation | Taking actions that adapt your program to ensure its ongoing effectiveness | My school adapts or changes the scheduling of physical activity each week as needed (e.g., if PE equipment is damaged and cannot be used, heat wave etc). |
|  |  | My school has a process to proactively adapt the scheduling of physical activity to meet changes in needs of the school community (e.g., to include other school programs). |
|  |  | My school makes decisions about which physical activity components are ineffective and should not continue when scheduling physical activity (e.g., energizers, GoNoodle, running etc.) |
| Organisational capacity | Having the internal support and resources needed to effectively manage your program and its activities | School systems (e.g., space, time allocation) are in place to support the scheduling of physical activity. |
|  |  | There are adequate resources and infrastructure within the school to schedule physical activity. |
|  |  | School executives manage staff and other resources effectively to ensure that the scheduling of physical activity is met. |
|  |  | My school has enough trained school champions to support the scheduling of physical activity. |
|  |  | School champions and teachers at my school have enough supervision and support to implement the scheduling physical activity. |
| Communications | Strategic communication with stakeholders and the public about your program | My school has communication strategies in place to secure and maintain our school communities’ support for scheduling physical activity. |
|  |  | Staff members at my school communicate the need for scheduling physical activity to the community (e.g., parents). |
|  |  | My schools’ scheduling of physical activity increases community awareness of the need for physical activity in children. |
| Program evaluation | Assessing your program to inform planning and document results | My school has a system in place to actively evaluate the scheduling of physical activity (e.g., improvements in children’s physical activity, student on-task behaviour etc.) |
|  |  | My school reports the outcomes of scheduling the recommended minutes of physical activity (e.g., Improvement in student physical activity levels). |
|  |  | Evaluation results inform the planning and implementation of the scheduling of physical activity. |
| Funding stability | Establishing a consistent financial base for your program | The school takes action to ensure there are ongoing funds to support the scheduling of physical activity (e.g., included in annual school budget, funding from P&C). |
|  |  | My school has a process in place to allow staff to attend professional development on scheduling physical activity (i.e., funding for ongoing professional development). |
|  |  | My school provides time at work for staff to plan their schedule for meeting the recommended minutes of physical activity. |
|  |  | My school can access a variety of funding sources to help schedule physical activity. |
