# Supplementary material for: School-level factors associated with the sustainment of weekly physical activity scheduled in Australian elementary schools: an observational study
Source: BMC Public Health. 2022 Jul 23;22:1408. doi: 10.1186/s12889-022-13732-6 (PMC9308175; doi:10.1186/s12889-022-13732-6)
Supplement: Supplementary file 3 — Additional file 3. Associations between factors and difference in weekly minutes of PA and energisers implemented between follow-up T1 and T2 for teachers who completed all time points. [file 12889_2022_13732_MOESM3_ESM.docx]

**Additional file 3.** Associations between factors and difference in weekly minutes of PA and energisers implemented between follow-up T1 and T2 for teachers who completed all time points

| **Factor** | **Total weekly minutes of PA implemented** | | | | **Total weekly minutes of energisers implemented** | | |
| --- | --- | --- | --- | --- | --- | --- | --- |
|  | Total (n) | Unadjusted coefficient (95%CI)^a^ | Adjusted coefficient (95%CI)^a^ | p-value^b^ | Unadjusted coefficient (95%CI)^a^ | Adjusted coefficient (95%CI)^a^ | p-value^b^ |
| ***School-level sociodemographic factors*** | | | | | | | |
| School size | 66 | -0.03 [-0.17; 0.11] | -0.04 [-0.18; 0.11] | 0.59 | -0.02 [-0.09; 0.05] | -0.02 [-0.09; 0.06] | 0.61 |
| School SEIFA  Most disadvantaged  Least disadvantaged (R1)^ | 66 | -8.57 [-53.32; 36.18] --- | -9.48 [-57.33; 38.38] --- | 0.67 | -6.02 [-29.10; 17.05] --- | -6.40 [-30.46; 17.67] --- | 0.58 |
| School remoteness  Major cities  Inner / outer regional /  remote Australia (R1)^ | 66 | -11.35 [-56.18; 33.47] --- | -11.10 [-59.62; 37.43] --- | 0.60 | -10.18 [-33.05; 12.68] --- | -10.15 [-33.98; 13.68] --- | 0.36 |
| School type  Catholic  Government (R1)^ | 66 | 30.25 [-15.69; 76.19] --- | 34.27 [-14.58; 83.11] --- | 0.16 | 22.81 [1.55; 44.08] --- | 24.17 [2.02; 46.33] --- | 0.06 |
| ***Teacher-reported school-level factors*** | | | | | | | |
| School PA plan or policy  Yes  No (R1)^ | 57 | -6.24 [-73.05; 60.57] --- | -4.21 [-72.64; 64.22] --- | 0.90 | 1.74 [-29.49; 32.96] --- | 1.03 [-30.81; 32.86] --- | 0.95 |
| Strategic planning | 65 | -8.68 [-25.45; 8.10] | -10.33 [-28.61; 7.94] | 0.26 | -8.03 [-16.04; -0.02] | -8.75 [-17.35; -0.16] | **0.046** |
| Environmental support | 66 | -6.99 [-26.10; 12.11] | -9.02 [-29.95; 11.90] | 0.39 | -5.10 [-14.15; 3.94] | -4.64 [-14.47; 5.19] | 0.35 |
| Program adaptation | 66 | -10.11 [-44.46; 24.23] | -2.85 [-23.99; 18.29] | 0.79 | -4.98 [-14.19; 4.24] | -4.66 [-14.59; 5.26] | 0.35 |
| Organisational capacity | 66 | -0.10 [-20.70; 20.51] | -0.70 [-23.96; 22.56] | 0.95 | -7.13 [-16.60; 2.35] | -7.37 [-17.86; 3.12] | 0.17 |
| Communications | 66 | -6.48 [-24.14; 11.18] | -8.91 [-28.87; 11.05] | 0.38 | -7.35 [-15.65; 0.95] | -7.81 [-17.11; 1.49] | 0.10 |
| Program evaluation | 66 | -11.23 [-24.89; 2.42] | -12.12 [-26.91; 2.66] | 0.11 | -7.17 [-13.61; -0.72] | -7.39 [-14.29; -0.50] | **0.036** |
| Funding stability | 66 | 4.43 [-13.88; 22.74] | 5.17 [-14.56; 24.90] | 0.60 | -5.64 [-14.08; 2.79] | -5.40 [-14.19; 3.39] | 0.22 |
| ^a^Linear mixed regression analyses were used to calculate coefficient and p-values. Output is for teachers who completed all time points (i.e., baseline, follow-up T1 and T2). Coefficients correspond to the influence of a unit increase in each factor on the difference in PA and energisers implemented across the school week between 12 and 18-month follow-up. Results are reported as adjusted and unadjusted regression coefficients with corresponding 95% confidence intervals. Adjusted analyses included a random intercept for school and were adjusted for number of years teaching, whether they are a PE teacher and whether they are employed full time.  ^R1 indicates the reference group for dichotomous variables.  ^b^p-value listed is for the coefficient from the adjusted model. Significance was set at p ≤ 0.05 with bolded p-values indicating significance. | | | | | | | |
